# Is the Study of Biology of Advantage to the Practical Dentist?

**Published:** 1912-02-15

**Authors:** C. M. Wright

**Affiliations:** Cincinnati, Ohio


### IS THE STUDY OF BIOLOGY OF ADVANTAGE TO THE PRACTICAL DENTIST?

BY C. M. WRIGHT, D.D.S., CINCINNATI, OHIO.

This question is asked almost daily by the dental student and by the busy practitioner.

It is not always asked in these exact words. One may have in mind Physiology, another Histology, another any subject in medical science, instead of the term "Biology." Biology includes them all.

By Biology I mean the science which treats of living matter in contra-distinction to the sciences which deal with non-living matter, such as metals, minerals and wood.

The study of Biology is the study of organisms, i. e., of live creatures, like the amoeba, composed of a single cell, a so-called simple organism; and, like man, a complex organism composed of uncounted millions of cells.

Biology includes the study of the live parts of a creature, —of the living tissues, bone, mucous membrane, dentin, muscle and nerve. It includes all that can be learned about living matter; about its morphology or structure, microscopic and macroscopic; about its natural activities in the condition we call health; about its minute and aggregated reactions to changed environment, and to different irritations; about its variations in function and structure in the condition we define as disease.

Living matter, wherever found, in the animal and vegetable world, and in fungi, is the subject investigated by the Biology, upon which the science of medicine rests. It is classified under the heads of Histology and Anatomy, Physiology, Bio-chemistry, Bacteriology and Pathology, and is the special section of this great science to which I refer in the question asked today.

Is the study of advantage to us practical dentists?

We spend hours and days at a lathe grinding porcelain; we file and scrape and polish vulcanized rubber; we adjust platinum matrices to cavities in teeth, and bake and rebake and enamel little bits of porcelain for stoppings for these cavities. We push and mallet and burnish gold into cavities in teeth with all the careful attention to joints, edges and surfaces that a jeweler must give to a piece of work at his bench. We sit and study plaster models of jaws and teeth for the purpose of inventing some mechanical appliance for expanding a maxillary arch and regulating mal-occlusion and mal-position of the teeth. This we do for esthetic as well as for functional reasons. In short, we give the best portion of our lives to mechanical work upon a number of mineral and metallic (non-living) materials in the daily practice of our profession. We have felt, too, that all this work with the hands was worthy of our most devoted efforts; that dentistry would not be dentistry without this continual daily application of the laws of physics and mechanics.

Is it any wonder, then, in view of all this hand work, that the question should at times arise in all seriousness, in each of our minds, "Of what advantage to me in my practice is a study of Biology?" or "What has Biology to do with all this technical work?"

Dentistry is a manual profession. It requires careful and tedious training of the hands. Those clusters of neurons which preside over the movements of the fingers and arms —the co-ordinated contractions and relaxations of muscle protoplasm of these organs—become highly developed in the dentist. The whole trend of his life-work favors the development of the mechanical qualities of brain and nerve and muscle. The problems which are presented to him every hour of his day must be solved by finger craft. His capital is ingenuity and skill. These are the assets which have helped to place American dentistry at the head of the profession of the world. We have become conspicuous and distinguished in every civilized country of the world on account of our marvelous skill and ingenuity, and not on account of our medical knowledge.

When the dentist forgets his cunning and loses his digital skill, and his interest in *how to do* things, dentistry will cease to be useful as a specialty. I insist upon this, notwithstanding the outbreak of pessimism in regard to the mechanics of our profession, indulged in lately by our learned friend, Dr. Talbot Even modern dental "Prophylaxis" implies skillful hand work. If we accept what I have recalled of the work and life of the dentist, why should we bother about Biology? What has Biology done for dentistry? Is there a sharply defined line of demarkation between "theory" and "practice?" We are in the habit, in speaking and writing, of taking this distinct line as a real thing.

A number of years ago I had the pleasure of meeting a distinguished German dentist in Freiberg, Baden, who had recently visited America and had been highly honored by the National Dental Association of this country. In relating to me the results of his study and observations in America, he said: "The American dentist has it here," and he held up his hand and wiggled his fingers. "the German dentist has it here," and he placed his forefinger on his forehead. This was an ocular and pantomimic illustration of what we oracularly mean by theory and practice. This might have ended the subject had not my esteemed friend added, "Now I have been in America and have it *here* and *here*" —fingers and head. I mildly hinted that modesty had lost its place in the scuffle. This caused my friend very much distress, and profuse explanations followed.

I have, in this broadly sketched picture, tried to present, as fairly as I can, the silver side of the shield. I have tried to make plain that one side is of silver, and that this side is necessary in the structure of our dental shield. Now, let us turn the shield and find, if we can, the gold on the other side.

Why do we call our vocation a *profession*—a scientic profession? Why do we, while working every day with our hands, just like any artisan, and fully acknowledging the fact that the skillful touch, the trained hand and the ingenious mind are the most prominent features of dentistry, know, deep down in our hearts, that we are separated by a wide and deep gulf from the best silversmith, jeweler and blacksmith? Why does the dentist know that he is not an artisan, but a professor of an art—a professional man?

Let me answer. It is because of the Biology that underlies and permeates and gives reasons for every single act of the dentist in his practice. We are fully armed in many ways to defend as a *profession* against those among us who, with cringing subserviency to their own false idea of the "medical profession," sneer at "mechanics." This is to me an unaccountable attitude for a dentist to assume, for it can be easily shown that the lights of the medical world —the most broadly cultured men in the mother profession, as well as the writers for the medical press—are sincere in their many public and private expresssions of high appreciation of the science, as well as of the art, of the dentist. Personally, I should go no further in her defense than by showing that dentistry, modern dentistry, is founded upon Biology.

One statement to sustain this proposition is, *that the dentist operates upon live matter—living tissue.* He carves and shapes living matter, specialized into threads, with antecellular substance arranged as dental tubes, every time he prepares the simplest cavity in a tooth for a filling. He selects and applies his materials for the filling itself with reference to its compatibility with the intertubular protoplasm of the tissue, and with regard to its prophylactic qualities in the hindrance of the recurrence of diseases produced by the specific action of well known bacteria. He performs this most delicate and accurate surgery upon histological elements, the origin of each one of which he can trace back to an embryonic layer in the blastoderm of an oven.

Filling a tooth is a common, every-hour operation of the dentist, and yet it is really what we know it to be, minute surgery and not mechanic's work, for the entire surgical area quivers with life. That live dentin is as much a part of the live man as is his brain, his arm, or his liver. That live dentin gets its share of the beefsteak taken in by the complex organism, man, as food, as do the liver and the brain their shares, and the metabolism of the one is the same as in the others. If we could by some exquisitely accurate instrument (such as Black might construct) measure the energy, the kinetic energy, liberated by this metabolism in a single protoplasmic arm of one odontoblast in a single denturial tubule, we should notice variations, depending upon the quantity and quality of lymph supplied to it as food; and variations, when we cut across it with our excavator or brew, in its response to or reaction against a mechanical imitatation; and we cut millions of these living processes in every operation for the preparation of a cavity for a filling.

This proposition may at first glance seem rather far-fetched, but it is not, and is absolutely in accordance with the laws of living matter while alive, and with the facts which the biologist has discovered in regard to every bit of live matter, whether found in animals, plants or fungi.

The surgeon, when he operates upon living matter, has a picture in his mind of the efforts for repair and regeneration which will be made by the neighboring live cells of each different tissue which he must sever. He sees the blood and lymph hurriedly responding to calls from the nerves bringing with them materials and means for arresting the hemorrhage, for cleaning the wound, for smoothing the ragged edges of supporting tissues, and for erecting scaffolding for temporary use, until the different tissue cells can proceed more deliberately to rebuild the parts after the original plan. The dentist has the same picture in his mind when he operates. The man who does not have this picture in his mind when he touches living matter in any surgical way is the mechanic, no matter how skillfully he may wield an instrument.

I said a moment ago that skill and ingenuity, and not medical knowledge, were the prime assets of the American dentist. Skill and ingenuity are the assets of the great surgeon, and dentistry and surgery are built upon Biology.

The dentist as well as the surgeon must have assets that are immediately available, and skill and ingenuity make these; but we need not be told that the skill and ingenuity of the modern surgeon and dentist would not exist, could never have been acquired, without the labors in experimentation and investigation of the biologist—the anatomist; the physiologist; the bacteriologist and the pathologist.

The skillful dentist of today is deeply indebted to the biologists of the past, and of the present. If we only look backward to those anatomists and physiologists who are called the fathers of modern surgery we shall begin to realize the amount of our indebtedness. John Hunter, for instance, was born only 180 years ago. Pasteur and Chauveau suggested an explanation for the phenomenon of acquired immunity only about thirty years ago. Metchnikoff startled the world with his theory of phagocytosis only twenty-five years ago. Ehrlich published his epoch-making theory on the side chain (seitenkeltentheovis) about twelve years ago, and the dentist has been as keen in acknowledging and appreciating the labors of these and other biologists as have practical physicians and surgeons.

The leaders in modern dentistry today are not only skillful operators but good diagnosticians. They know *how* to operate and they know *why* they operate. This "why" has been gained by a deeper study of the fundamental principles of Biology.

This knowledge of Biology stimulates and awakens the "pathological sense." Now, while we admit that our entire superstructure rests on Biology, I am well aware that the busy dentist, wearied with his labors on the building itself —the visible portion which is above ground and which stands out like a skyscraper, a grand sight—often fails to look after the foundations,—the portion which seems to be shrouded in mystery. He is too tired to go down in the cellar and subcellar and satisfy himself of the soundness, the strength, and, I will add, the beauty of this basal portion of the building. Some hesitate to go down because they have almost forgotten the way since their college days. Would it be presumptive if I should try at this time to direct these to the cellar?

First: Get out your modern text-book on Histology or Physiology, and give your attention at odd times for some weeks to a thorough study of the structure and properties of protoplasm—living matter. Study the life history of a simple celled organism, like the amoeba. Don't attempt to go down one step further until the little creature is so familiar to you that you can see it and recognize its vital phenomena in your mind's eye whenever you think of a lung or kidney, a brain or a liver, a muscle or a bone, and can cry out, "Hello! Here you are, Mr. Amoeba (specialized as a neuron, or as a bone cell, or as an odontoblast), living the same life in relation to food, waste and energy (though the manifestations of energy vary) that you did when you were free from your present special occupation, and unaffected by your present special environment." A little mass of live matter, with all the innate qualities of metabolism, of imitability, of contractility, and of reproduction.

Second: After acquiring this familiarity, look up the different kinds of elemental tissues of the body. Look up the localities and surroundings of epithelial tissue, of the several varieties of connective tissues, of muscle and nerve tissue.

Third: After observing all the points in the little fellow's healthy actions and reactions on these different locations of the body, study the effects of bad air, bad food, cold, heat, light, poisons of various sorts and mechanical injuries, upon these small bits of life. Study the bacterial enemies of our small cell, and observe the powers of resistance it possess in itself. This is the pathology of it.

I need go no further, for after this the way becomes easy. This is the most direct route to the subcellar of our building. We may now continue our strides and go on to investigations into physiological chemistry and pathological morphology, and the practical application of therapy and prophylaxis.

This study gives an immense amount of satisfaction, and to the tired dentist it gives a pleasure greater than can be had from Galoreaux or Conan Doyle.

It also awakens gratitude toward every investigator in the different departments of Biology; and, what is particularly valuable, it inspires us with a higher opinion of and a deeper respect for our daily work. We become optimistic and enthusiastic, practical dentists, with never a regret that we have not annexed to our names the letters "M. D." by early studies in a medical college, or by the courtesy of some struggling and possibly irregular medical school; for we know that even the title "D. D. S." can be only of real value and meaning to the persistent student in this, our department of medicine.

I added this last sentence or two to the paper while on the train, because of a little talk with Dr. McGee, who has lately come from Richmond, Va., and taken a teacher's position, a professorship, in the Ohio College of Dental Surgery.

He told me yesterday of the efforts of a number of the members of the profession in Virginia to make the medical degree, the M. D., a qualification for entrance to a dental college.

From my standpoint and viewpoint such an act would be the death blow to dentistry. It would be an act favoring degeneration, and not normal, healthy development. We have reached our present position of honor among the specialists of medicine by a determined, independent and honorable work by many men whom we are proud to enshrine in our memories. Our traditions we hold as a sacred inheritance. By losing our identity, our distinct and unique position, and becoming a fragment, a ragged edge of the medical profession, we should soon feel that we had indeed sold our birthright for a mess of pottage.

The practice of medicine cannot prepare a man for the practice of dentistry any more than the practice of dentistry prepares a man for the practice of medicine. Yet we are as free today as we have been from the beginning of the history of dentistry to absorb every new discovery or doctrine in general pathology, in connection with the modern specialist, general physician or surgeon. For, as I have tried to show in this little paper, dentistry is securely founded upon the principles of Biology.

Two distinct buildings—one the art of the physician and surgeon and the other the art of the dentist—have been reared on the same foundation. Each has its separate college.—*Dental Summary.*

